# Awake prone position in COVID-19-related acute respiratory failure: a meta-analysis of randomized controlled trials

**DOI:** 10.1186/s12890-023-02442-3

**Published:** 2023-04-26

**Authors:** Sun Qin, Wei Chang, Fei Peng, Zihan Hu, Yi Yang

**Affiliations:** Jiangsu Provincial Key Laboratory of Critical Care Medicine, Department of Critical Care Medicine, School of Medicine, Zhongda Hospital, Southeast University, Nanjing, 210009 China

**Keywords:** COVID-19, Hypoxemic respiratory failure, Intubation, Awake prone position

## Abstract

**Background:**

We aimed to investigate the effects of awake prone positioning (APP) in nonintubated adult patients with acute hypoxemic respiratory failure due to COVID-19.

**Methods:**

The PubMed, Embase, Web of Science and Cochrane Central Register databases were searched up to June 1, 2022. All randomized trials investigating the effects of APP were included in the present meta-analysis. The primary outcome was intubation rate, and the secondary outcomes included the length of intensive care unit (ICU) stay, hospital stay, and mortality. Prescribed subgroup analysis was also conducted.

**Results:**

A total of 10 randomized trials enrolling 2324 patients were ultimately included in the present study. The results indicated that APP was associated with a significant reduction in the intubation rate (OR 0.77, 95% CI 0.63 to 0.93, *P* = 0.007). However, no differences could be observed in the length of ICU stay or hospitalization or mortality. Subgroup analysis suggested that patients in the ICU settings (OR 0.74, 95% CI 0.60 to 0.91, *P* = 0.004), patients whose median APP time was more than 4 h (OR 0.77, 95% CI 0.63 to 0.93, *P* = 0.008), and patients with an average baseline SpO_2_ to FiO_2_ ratio less than 200 (OR 0.75, 95% CI 0.61 to 0.92) were more likely to benefit from APP, indicated a significantly reduced intubation rate.

**Conclusion:**

Based on the current evidence, nonintubated adult patients with hypoxemic respiratory failure due to COVID-19 infection who underwent APP were shown to have a significantly reduced intubation rate. However, no differences in ICU or hospital length of stay or mortality could be observed between APP and usual care.

**Registration number:**

CRD42022337846

**Supplementary Information:**

The online version contains supplementary material available at 10.1186/s12890-023-02442-3.

## Background

Prone position is widely used in severe acute respiratory distress syndrome (ARDS) patients since its effects on improving oxygenation and mortality have been proven [1, 2]. The awake prone position (APP), which refers to the prone position for nonintubated patients, was attempted for patients with acute respiratory failure many years ago [3], and its potential effects in avoiding intubation have been shown in a recent study [4].

During the COVID-19 pandemic APP has been broadly used worldwide and incorporated into clinical guidelines and expert consensus statements [5, 6]. Multiple previous observational studies reported improved oxygenation with APP among patients with COVID-19-related acute respiratory failure, but none of them demonstrated a benefit in avoiding intubation or reducing mortality [7]. Since the last year, some randomized controlled studies have tried to determine whether APP could reduce intubation and mortality, but controversy still exists. In August 2021, an international randomized controlled meta-trial that included more than 1100 patients showed that APP significantly reduced the intubation rate for patients with COVID-19-related respiratory failure requiring high-flow nasal cannula support [8]. A recently published meta-analysis including ten RCTs (1985 patients) and 19 observational studies (2669 patients) also supported APP in reducing intubation need [9]. However, Alhazzani and colleagues’ study [10] overturned this conclusion since their study did not find benefits of the prone position in reducing intubation and improving outcomes.

Considering the controversy of APP in clinical use, we conducted this meta-analysis to evaluate the effects of APP on intubation and all-cause mortality in patients with COVID-19-related acute hypoxemic respiratory failure. Essential to this is to find specific subpopulations who are likely to benefit most from awake prone positioning. We believe these findings could help clinicians in their daily practice and maximize the benefits for patients with COVID-19 during the ongoing pandemic.

## Methods

This study was prepared in accordance with the Preferred Reporting Items for Systemic Reviews and Meta-Analyses (PRISMA) statement (Additional File 1), and our protocol was registered in the International Prospective Register of Systematic Reviews (CRD42022337846).

### Information sources

Two reviewers searched the PubMed, Embase, Cochrane Central Register and Web of Science database from Jan 1, 2020, to June 10, 2022. We included studies published in English; there were no limitations regarding the location of the study. When potentially relevant reviews or meta-analyses were found, a retrograde search was conducted to assess further studies.

### Search strategy

The following key words were introduced in the search: “prone position”, “awake or non-intubated”, “COVID-19” and “SARS-CoV-2”. The complete search strategy is provided in Additional File 2.

### Eligibility criteria

Trials that met the following criteria were included in this meta-analysis: [1] study population with acute hypoxemic respiratory failure due to suspected or confirmed COVID-19 infection, who were not intubated; [2] the intervention would be APP, with no restrictions on durations; [3] the control group of usual care, with no restrictions on positionings; [4] reported the outcomes of interest, and [5] study design would be randomized controlled trials. The exclusion criteria included: [1] in vitro or animal experiments; [2] pediatric or pregnant subjects; [3] nonrandomized study design; and [4] lack of results on outcomes of interest.

### Study selection

Titles and abstracts were initially reviewed separately by two reviewers. When potentially relevant studies were found, complete manuscripts were obtained for further review. All the studies were reviewed, assessed and selected by two separate reviewers, with any disputes solved by consensus or discussion with a third reviewer.

### Data items

The following data were extracted from the studies: [1] subject characteristics, including the demographic information of age and gender, the baseline oxygenations (evaluated by SpO_2_ to FiO_2_ or PaO_2_ to FiO_2_), the settings of the trials (ICU or ward); [2] details in interventions, including the daily and overall duration of prone position, and [3] outcomes of interest. The primary outcomes were the intubation rate, and the secondary outcomes were clinical parameters, including the improvement of oxygenations if available, the length of ICU stay and hospitalization and mortality. The SpO2/FiO2 to PaO2/FiO2 conversion formula was used as previously reported, as SpO_2_/FiO2 = 64 + 0.84×(PaO2/FiO2) [11].

### Risk of bias assessment

The internal validity and risk of bias of the enrolled studies were evaluated by two independent reviewers using the Cochrane Collaboration’s Handbook protocols [12]. The risk of bias of the studies was assessed as “Yes”, “No” or “Unclear” following scrutinization of the methods and procedures taken in the articles.

### Summary measures

Continuous variables were presented as the means with standard deviations (SD) and converted if medians with interquartile ranges (IQR) or 95% confidence intervals (CI) were used; and were compared by mean difference (MD) or standard mean difference (SMD) according to the uniformity of the units of the parameters. Categorical variables were described as frequencies or proportions and were estimated by odds ratios (ORs) with 95% CIs.

### Statistical analysis

The data obtained from the enrolled studies were calculated using Review Manager 5.4 (The Nordic Cochrane Centre, Cochrane Collaboration, Copenhagen). Mantel-Haenszel statistics were used for dichotomous variable measurements, and an inverse variance model was applied for continuous variable comparisons. A random-effects model was used in case of significant heterogeneity. Cochrane I^2^ statistics was applied to evaluate the heterogeneity between studies, and I^2^ > 50% was defined as high heterogeneity. For sensitivity analysis, each study was sequentially removed with the remaining dataset reanalyzed to calculate the statistical significance or favoring directions to evaluate the robustness of the results.

Univariate meta-regression involving the factors of study design and performance was used to find the potential sources contributing to heterogeneity between studies. The prescribed potential factors included the sample size, overall intubation rate, APP duration and setting (ICU).

Prescribed subgroup analysis dividing the studies by average baseline oxygenations evaluated by PaO_2_ to FiO_2_ ratio or SpO_2_ to FiO_2_ ratio [11]; the average duration of APP; and the different settings of the studies including ICU settings and non-ICU settings.

## Results

### Study selection and characteristics

**Fig. 1:**
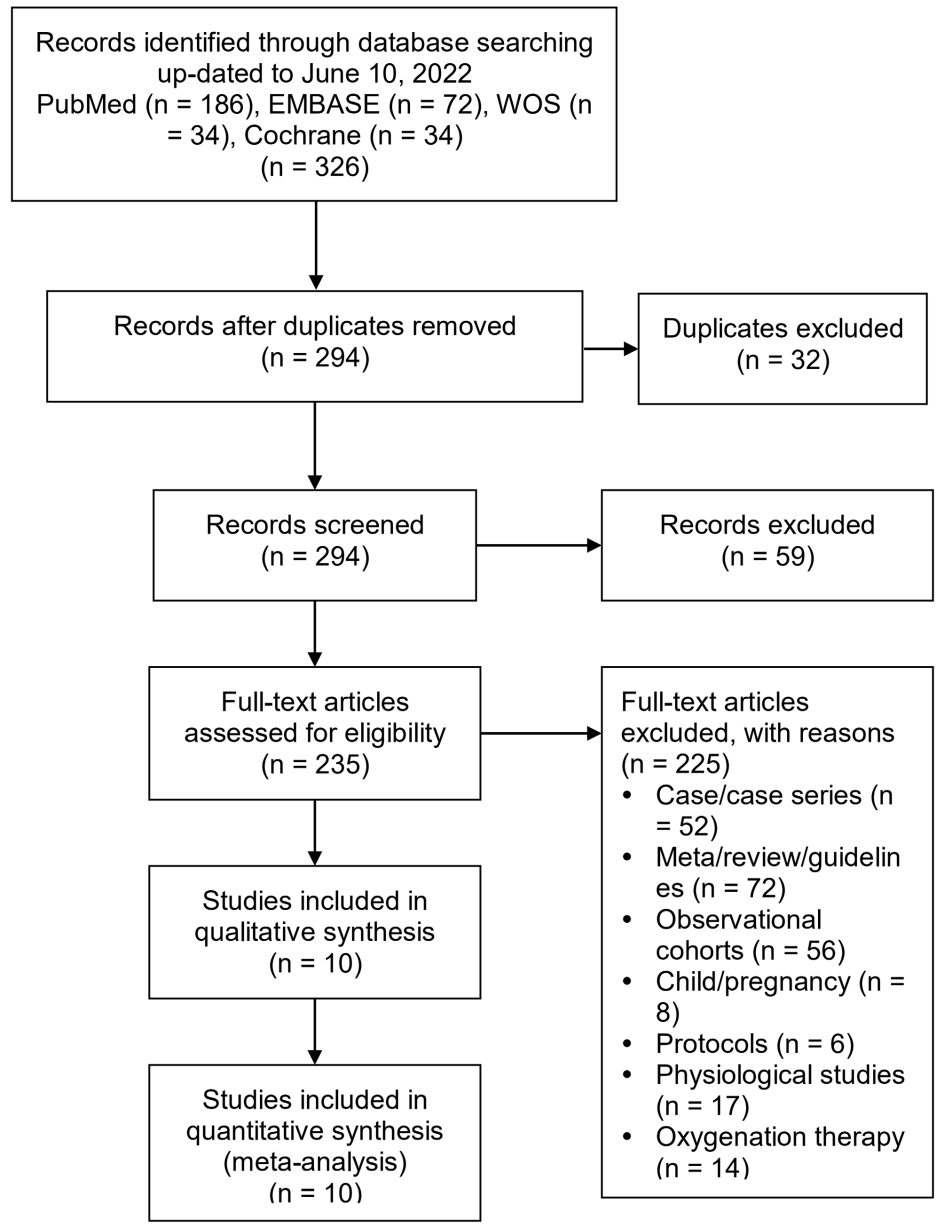
Study flow diagram Figure 1 is incorrect. We will up-load the correct figure.

We identified a total of 326 articles with our search strategy. We screened 294 titles and abstracts after 32 duplicates were removed. A total of 10 randomized controlled trials enrolling 2324 patients were included in this meta-analysis [8, 10, 13–20] (Fig. 1). The detailed risks of bias assessment of the enrolled trials were described in Additional File 3.

Among the 10 included studies, 5 studies were set in the ICU [8, 10, 14, 15, 18], and 5 studies were in the general wards [13, 16, 17, 19, 20]; the final overall intubation rate ranged from zero to 66.7% [18]; the reported baseline SpO2 to FiO2 ratio varied from less than 150 [8, 10] to more than 300 [13, 17, 20]; and the APP procedures were variable, with the average daily duration of APP varying between less than 1 h to approximately 9 h. The characteristics of the enrolled trials were presented in Table 1.

**Table 1:** Characteristics of the enrolled studies. APP awake prone position, NR not reported, SpO_2_ transcutaneous oxygen saturation, FiO_2_ inspired oxygen fraction. SpO_2_ to FiO_2_ ratio was estimated by following formula S/F = 64 + 0.84×(P/F) if P/F were reported in the study

Study	Settings	Patient Number	Estimated baseline S/F, mmHg	Overall intubation rate, %	Daily APP duration, hour
Alhazzani 2022	ICU	400	133.9 ± 52.5	37.3	4.8 (1.8 to 8.0)
Ehrmann 2021	ICU	1121	148.2 ± 43.5	36.4	5.0 (1.6 to 8.8)
Fralick 2022	Non-ICU	250	303.9 ± 18.4	4.4	6 (1.5 to 12.8)
Gad 2021	ICU	30	163.45 ± 109.9	20.0	each session last for 1 to 2 h according to patients to tolerability with 3-hs apart during waking hours.
Jayakumar 2021	ICU	60	226.1 ± 164.8	13.3	a minimum of 6-h in a day
Johnson 2021	Non-ICU	30	NR	6.7	1.6 (0.2 to 3.1)
Kharat 2021	Non-ICU	27	329.3 ± 56.0	0.0	4.9 ± 3.6
Rampon 2022	Non-ICU	293	396 (306–387)	2.0	NR
Rosen 2021	ICU	75	160 ± 87.5	66.7	9.0 ± 4.6 (control group, 3 h)
Taylor 2021	Non-ICU	40	NR	0.0	10 to 120 min per day

NR, not reported, S/F = 64 + 0.84×(P/F)(12)

### Synthesis of results

#### Primary outcomes

**Fig. 2:**
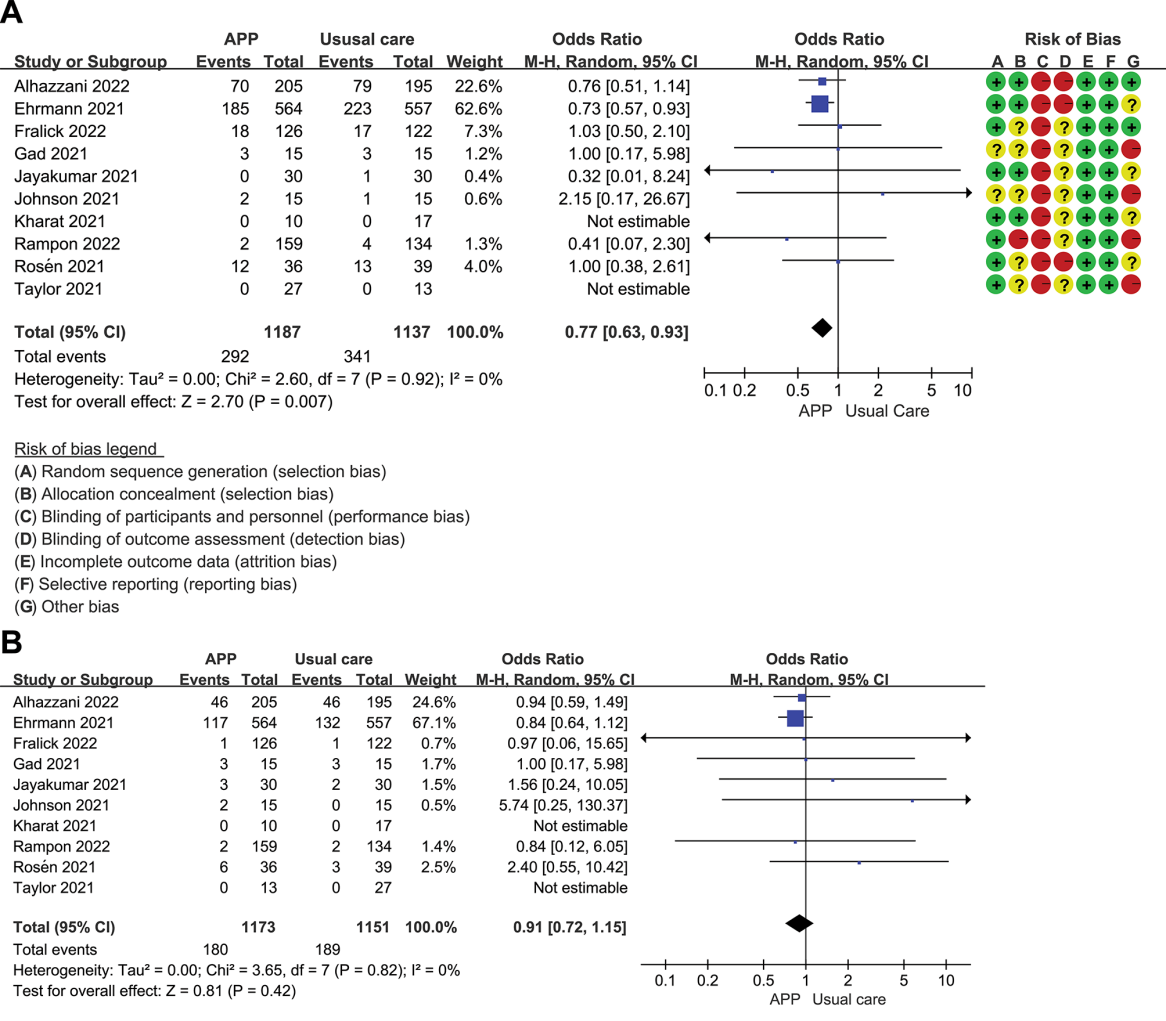
Intubation rate (**A**) and all-cause mortality (**B**) for included randomized controlled trials

All ten studies reported patients requiring intubation as an outcome and the results revealed a significant decrease in the APP group compared with the control group, with an OR of 0.77 (95% CI 0.63 to 0.93, *P* = 0.007) (Fig. 2A).

#### Secondary outcomes

We compared the clinical outcomes between the APP group and the control group and found that APP decreased neither ICU length of stay (*P* = 0.40) nor length of hospital stay (*P* = 0.14) (Additional File 4). There was no significant difference between the APP group and the control group in all-cause mortality (*P* = 0.42) (Fig. 2B).

### Subgroup analysis

**Fig. 3:**
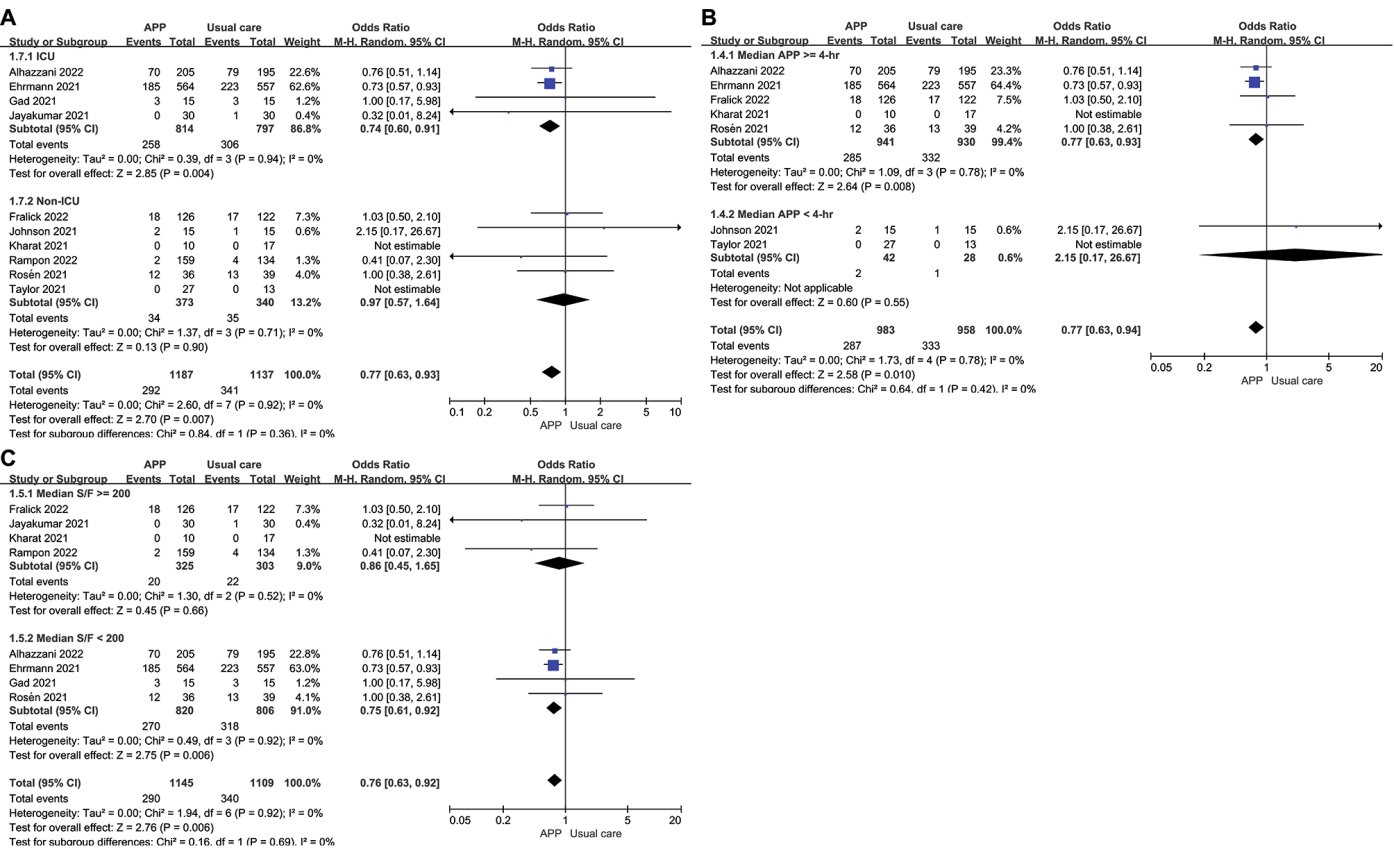
Subgroup analysis of intubation. **A**. ICU vs. non-ICU, **B**. APP ≤ 4 h vs. > 4 h, **C**. Average baseline SpO_2_ to FiO_2_ ratio < 200 vs. ≥ 200

A prescribed subgroup analysis was deployed for further interpretation, and we found that patients with average baseline SpO_2_/FiO_2_＜200 (OR 0.75, 95% CI 0.61 to 0.92, *P* = 0.006) and patients who received an average accumulated APP of more than 4 h (OR 0.77, 95% CI 0.63 to 0.93, *P* = 0.008) were likely to benefit from APP, as indicated by a reduced intubation rate. In the subgroup of patients admitted to ICU, APP significantly decreased intubation rate (OR 0.74, 95% CI 0.60 to 0.91, *P* = 0.004) (Fig. 3).

### Risk of bias and sensitivity analysis

Both fixed and random effects models were used to test the results, and we found no change in statistical significance. Each individual study was removed with the remaining studies analyzed, and we found that the statistical significance was obliviated when the study by Ehrmann et al. [8] was removed from the analysis, which suggested that the result might not be robust (Additional File 5). Visual inspection of the funnel plots revealed asymmetry in the distribution of the results, which suggested possible publication bias of the studies. We used the trim and fill method to further test the publication bias, and there were no changes in the results, suggesting no significant publication bias (Additional File 6).

### Meta-regression

Although no significant heterogeneity could be observed by I^2^ statistics (I-square = 0), a meta-regression analysis was conducted. However, we did not find factors that were potentially associated with heterogeneity between studies, including the sample size, the number of sites, the overall intubation rate, the average hour of APP, and whether the patients were in the ICU setting or general wards (Additional File 7).

## Discussion

In the present meta-analysis, we included ten trials enrolling 2324 patients with COVID-19-related acute hypoxemic respiratory failure and found that APP significantly decreased the intubation rate. Our findings were consistent with a recently published meta-analysis [9], which included ten RCTs that included a total of 1985 patients and 19 observational studies that included a total of 2669 patients. However, a recent RCT implemented in 21 hospitals with 400 patients overthrown this conclusion [10]. Our study validated the effect of APP in reducing the intubation rate for COVID-19-related respiratory failure patients.

The findings of the subgroup analysis showed a specific group of COVID-19-related respiratory failure patients who can benefit from APP. Those who received APP treatment for more than 4 h or those whose baseline SaO_2_/FiO_2_＜200 were more likely to avoid intubation. Patients in the ICU were also tended to benefit from APP. However, in this meta-analysis, we used an average APP time, so the patients in the individual group may not be precisely APP for the appointed time, and a patient-level meta-analysis may solve this issue.

For moderate to severe ARDS patients using invasive mechanical ventilation, the prone position was a very important treatment that can significantly improve patient outcomes [2]. Subsequent studies found that the prone position was a time-dependent therapy, which meant that only when the duration of prone position was more than 12 h, did its effects begin to be revealed [21]. A recent physiological study on the prone position in mechanically ventilated ARDS patients also proved that only after a period of prone position did ventilation–perfusion matching and oxygenation improve [21]. For COVID-19-related respiratory failure patients, the pathophysiological changes during the awake prone position might be the same and there was also a threshold duration time of APP. Our results showed that when the duration of APP exceeded 4 h within one day, its benefits in avoiding intubation were revealed. Considering the comforts, risks and compliance of APP for COVID-19 patients, a threshold of 12 h for prone position treatment is not applicable for APP. This subgroup analysis of awake during time may explain the negative results in some studies and provide a new perspective in future studies and more relevant data are needed to establish an appropriate duration goal for APP [22].

We also compared the patients in the ICU settings and those in non-ICU settings and found that the patients in the ICU settings were significantly associated with a decreased intubation rate, which we thought, could be explained by the following reasons. First, the patients enrolled in the ICU may have had more severe disease, and they were more likely to progress to intubation than non-ICU patients. Second, the patients in the ICU were related to intensive monitoring, had higher nursing-to-patient ratios, and their adherence to APP was higher.

The main mechanisms of the prone position in improving of ARDS patients’ condition include increasing end-expiratory lung volume, decreasing alveolar shunt, decreasing tidal hyperinflation of the ventral regions and promoting the recruitment of the dorsal regions of the lung, which leads to better ventilation-perfusion matching [23]. For COVID-19 related respiratory failure patients who received APP treatment, all these mechanisms exist [24]. Moreover, awake patients with spontaneous breathing during prone positioning could experience improved gas exchange, decreased inspiratory effort and lung stress, and an attenuated systemic inflammatory response [25]. A recent study also demonstrated that for patients with COVID-19-induced acute hypoxemic respiratory failure (AHRF) and treated by high flow nasal cannula oxygen therapy (HFNC) and APP, APP was associated with improvements in the aeration of the dorsal lung zones [26]. These factors might explain the effects of the awake prone position in avoiding intubation.

However, there were many bias factors such as the duration of awake prone position, the compliance of this treatment and the severity of included patients in recent studies about APP. Moreover, some researchers found that after three days of APP treatment lung aeration only improved in COVID-19 patients who eventually avoided intubation/death [27], which meant there were individual differences in patients’ responses to APP. Another issue about the lack of difference in mortality was that for patients receiving APP treatment, the improved oxygenation might conceal deterioration of the patients’ condition. A portion of those patients received delayed mechanical ventilation, which would result in worse outcomes [28]. Further studies on the safety interval of APP treatment and its effects on mortality are warranted.

In our meta-analysis, ten randomized controlled trials were included in this paper, and the sample sizes of 2 studies were significantly larger than those of other studies, which may have led to heterogeneity [8, 10]. Sensitivity analysis shows that the study of Ehrmann et al. has a great influence on the results, which can be explained partly by its relatively large sample size. The studies from Ehrmann et al. and Alhazzani et al. were the top two studies with large sample sizes. However, the primary outcome varied between the two studies. In Ehrmann’s study, intubation criteria were specific, and one of the intubation criteria was that severe hypoxemia with SpO2 below 90% despite an FiO_2_ of ≥ 0·8. We did not find specific intubation criteria in Alhazzani’s study, but for patients included in Alhazzani’s study, the interquartile range of FiO_2_ was 52–90, which indicated that a small number of patients had already met the intubation criteria from Ehrmann’s research. We think that different intubation criteria may be one reason for the different results.

This study had several limitations. Regarding the nature of the enrolled studies, the APP performance could not be blinded to the subjects or investigators, which would potentially bring bias to the results; two of the included studies reported no intubation or mortality, which did not contribute to the pooled results; we used the average value of the included studies for the subgroup analysis, and a patient-level meta-analysis may be warranted.

## Conclusion

Based on the current evidence, nonintubated patients with acute hypoxemic respiratory failure due to COVID-19 who underwent APP treatment were associated with a significantly increased intubation rate. However, no differences in ICU or hospital length of stay, or mortality could be observed between APP and usual care. Subgroup analysis suggested that patients admitted to the ICU and with a lower SpO_2_-to-FiO_2_ ratio were more likely to benefit from APP.

## Electronic supplementary material

Below is the link to the electronic supplementary material.

Supplementary Material 1

Supplementary Material 2

Supplementary Material 3

Supplementary Material 4

Supplementary Material 5

Supplementary Material 6

Supplementary Material 7

## Data Availability

The datasets used and/or analyzed during the current study are available from the corresponding author upon reasonable request.

## Abbreviations

APP: Awake prone position
ICU: Intensive care unit
OR: Odds ratio
CI: Confidence interval
SpO2: Transcutaneous oxygen saturation
FiO2: inspired oxygen
ARDS: Acute respiratory distress syndrome
RCT: Randomized controlled trial
PRISMA: Preferred Reporting Items for Systemic Reviews and Meta-Analyses
PaO2: Partial pressure of arterial oxygen
SD: Standard deviations
IQR: Interquartile ranges
MD: Mean difference
SMD: Standard mean difference
AHRF: Acute hypoxemic respiratory failure
HFNC: High flow nasal cannula oxygen therapy.
