# Supplementary material for: Awake prone position in COVID-19-related acute respiratory failure: a meta-analysis of randomized controlled trials
Source: BMC Pulm Med. 2023 Apr 26;23:145. doi: 10.1186/s12890-023-02442-3 (PMC10131466; doi:10.1186/s12890-023-02442-3)
Supplement: Supplementary file 2 — Supplementary Material 2 [file 12890_2023_2442_MOESM2_ESM.pdf]

Additional File 2: Search strategy

(prone position) AND (awake OR non-intubated) AND (COVID-19 OR SARS-CoV-2)
