# Supplementary material for: Awake prone position in COVID-19-related acute respiratory failure: a meta-analysis of randomized controlled trials
Source: BMC Pulm Med. 2023 Apr 26;23:145. doi: 10.1186/s12890-023-02442-3 (PMC10131466; doi:10.1186/s12890-023-02442-3)
Supplement: Supplementary file 3 — Supplementary Material 3 [file 12890_2023_2442_MOESM3_ESM.pdf]

Additional File 3: Detailed assessment of bias of the included articles

Alhazzani 2022

Bias	Authors' judgement	Support for judgement
**Random sequence generation (selection bias)**	**LOW**	***Quote*** "…Using a web-based randomization system with undisclosed variable block sizes of 2, 4, and 6, allocation was 1:1 and stratified by hospital and by ratio of oxygen saturation…" ***Comments*** This random sequence generation was validated and was not likely to bring bias to the results.
**Allocation concealment (selection bias)**	**LOW**	***Quote*** "…Concealment of randomization will be ensured at each center through a remote dedicated online randomization system…" ***Comments*** The allocation of concealment was validated and was not likely to bring bias to the results.
**Blinding of participants and personnel (performance bias)**	**HIGH**	***Quote*** "…the nature of the intervention precluded blinding of participating patients, families, the health care team, or research staff…" Comment The nature of the study impeded the blindness to the participants and investigators.
**Blinding of outcome assessment (detection bias)**	**HIGH**	***Quote*** "…Given the nature of the intervention, it is not possible to blind the trial biostatistician to the study group for the interim or final analyses…" ***Comments*** The author stated that the analysis process was not blinded to the analyst and investigators.
**Incomplete outcome data (attrition bias)**	**LOW**	***Comments*** As for all the patients enrolled in this study, all the patients' outcomes were reported with no missing data.
**Selective reporting (reporting bias)**	**LOW**	***Comments*** All the primary and secondary outcomes described in the method section was reported in the result section with no selective reporting.
**Other bias**	**LOW**	Comments There was no obvious bias pertaining the study

Ehrmann 2021

Bias	Authors' judgement	Support for judgement
**Random sequence generation (selection bias)**	**LOW**	Quote "…A statistician not involved in patient recruitment generated the allocation sequence for each individual trial…Allocation concealment at randomization was ensured by an online randomization system or with on-site opaque sealed envelopes…"<br>Comments online randomization system ensured the validation of the randomization.
**Allocation concealment (selection bias)**	**LOW**	Quote "…Allocation concealment at randomization was ensured by an online randomization system or with on-site opaque sealed envelopes…"<br>Comments on-site opaque sealed envelope guaranteed the randomization was concealed from the participants and investigators.
**Blinding of participants and personnel (performance bias)**	**HIGH**	Quote "…By the very nature of the intervention and design, trial participants, care providers, outcome assessors, and data analysts could not be blinded to the intervention…"<br>Comments To the nature of the study, it was not possible to be blind to the investigators and participants.
**Blinding of outcome assessment (detection bias)**	**HIGH**	Quote "…By the very nature of the intervention and design, trial participants, care providers, outcome assessors, and data analysts could not be blinded to the intervention…"<br>Comments The study was not blinded to the data analysts.
**Incomplete outcome data (attrition bias)**	**LOW**	***Comments*** As for all the patients enrolled in this study, all the patients' outcomes were reported with no missing data.
**Selective reporting (reporting bias)**	**LOW**	***Comments*** All the primary and secondary outcomes described in the method section was reported in the result section with no selective reporting.
**Other bias**	**UNCERTAIN**	Comments This was a meta-trial, which was likely to have heterogeneity between studies, which would potentially bring bias to the study.

Fralick 2022

Bias	Authors' judgement	Support for judgement
**Random sequence generation (selection bias)**	**LOW**	Quote "…Central randomization will be stratified by hospital site and performed using an interactive web-response system…"<br>Comments Central randomization ensured the validity of the randomization, which was not likely to bring bias to the studies.
**Allocation concealment (selection bias)**	**UNCERTAIN**	Comments The authors gave no info on how the allocation was concealed
**Blinding of participants and personnel (performance bias)**	**HIGH**	Quote "…The study will be unblinded. It will be impossible to blind patients or physicians. Given the objective nature of the primary outcome and the need for pragmatic approaches in the current clinical situation, an independent, blind clinical events committee is not planed…"
**Blinding of outcome assessment (detection bias)**	**UNCERTAIN**	Comments The authors did not mention whether the assessors were blinded to the results.
**Incomplete outcome data (attrition bias)**	**LOW**	***Comments*** As for all the patients enrolled in this study, all the patients' outcomes were reported with no missing data.
**Selective reporting (reporting bias)**	**LOW**	***Comments*** All the primary and secondary outcomes described in the method section was reported in the result section with no selective reporting.
**Other bias**	**LOW**	Comments There was no obvious bias pertaining the study

Gad 2021

Bias	Authors' judgement	Support for judgement
**Random sequence generation (selection bias)**	**UNCERTAIN**	***Quote*** "…all patients…after randomization the patients divided into two groups…" ***Comments*** The authors stated the patients were randomised. However, the authors did not describe in detail how the randomization method which would bring bias to the study.
**Allocation concealment (selection bias)**	**UNCERTAIN**	***Comments*** As nor did the author give any information of how the allocation was concealed, we though this would also bring bias to the study.
**Blinding of participants and personnel (performance bias)**	**HIGH**	***Comments*** As for the design of the study, it was impossible to blind to the performers.
**Blinding of outcome assessment (detection bias)**	**UNCERTAIN**	***Comments*** The authors did not give information whether the assessment of outcome was blinded to the statisticians.
**Incomplete outcome data (attrition bias)**	**LOW**	***Comments*** As for all the patients enrolled in this study, all the patients' outcomes were reported with no missing data.
**Selective reporting (reporting bias)**	**LOW**	***Comments*** All the primary and secondary outcomes described in the method section was reported in the result section with no selective reporting.
**Other bias**	**HIGH**	***Comments*** At the baseline information of the patients, PaO2/FiO2 between treatment group and control group was not balanced [126 (88-164) vs. 111 (97 - 175), *P* = 0.036], which we thought would bring potential bias to the results.

Jayakumar 2021

Bias	Authors' judgement	Support for judgement
**Random sequence generation (selection bias)**	**LOW**	Quote "…Patients were randomized in blocks of 4 using a computerized random number generator…"<br>Comments Random sequence was generated by computer, which was not likely to bring bias to the result.
**Allocation concealment (selection bias)**	**LOW**	Quote "…Allocation was concealed using sealed opaque envelopes..."<br>Comments Allocation was concealed from the investigators and participants.
**Blinding of participants and personnel (performance bias)**	**HIGH**	Comments As for the design of the studies, it was not possible to be blinded to the investigators.
**Blinding of outcome assessment (detection bias)**	**UNCERTAIN**	Comments The authors gave no information whether the outcome assessment was blinded to the investigators or the statisticians.
**Incomplete outcome data (attrition bias)**	**LOW**	Comments Of all the included patients' data was reported with no missing data.
**Selective reporting (reporting bias)**	**LOW**	Comments The outcomes described in the method section was reported in the result section, with no missing data.
**Other bias**	**UNCERTAIN**	Quote "…Our study also has important limitations…onset of illness was not a criterion for inclusion. Some of these patients might have had illness for longer periods than others…"<br>Comments There would be potential unbalance in the severity of illness according to the study design, which would potentially bring bias to the study.

Johnson 2021

Bias	Authors' judgement	Support for judgement
**Random sequence generation (selection bias)**	**UNCERTAIN**	***Quote*** "… We conducted a nonblinded pragmatic randomized controlled trial in symptomatic patients hospitalized with suspected or laboratory-confirmed COVID-19…"<br>***Comments*** The authors did not describe the details of the randomization, which would bring potential bias to the study.
**Allocation concealment (selection bias)**	**UNCERTAIN**	***Quote*** "… We conducted a nonblinded pragmatic randomized controlled trial in symptomatic patients hospitalized with suspected or laboratory-confirmed COVID-19…"<br>***Comments*** The authors did not describe the method in detail of the allocation concealment, which would potentially bring bias to the study
**Blinding of participants and personnel (performance bias)**	**HIGH**	***Comments*** the study did not describe the protocol of how the blinding to the investigators was made. However, according to the nature of this study, it was not possible to be blinded to the investigators.
**Blinding of outcome assessment (detection bias)**	**UNCERTAIN**	***Comments*** The authors made no description on the blinding of outcome assessment, which would potentially bring bias to the study.
**Incomplete outcome data (attrition bias)**	**LOW**	***Comments*** all the patients' data was reported
**Selective reporting (reporting bias)**	**LOW**	***Comments*** all the outcomes described in the protocol was reported in the result section
**Other bias**	**HIGH**	***Quote*** "…Interim analysis revealed that protocol adherence was poor…"<br>***Comments*** According to the results section, the median prone time was short of 1.6 (0.2 to 3.1) hours, which was potentially bring bias to the study.

Kharat 2021

Bias	Authors' judgement	Support for judgement
**Random sequence generation (selection bias)**	**UNCERTAIN**	Quote "…Six clusters were selected and a computer-generated randomization scheme was used to assign each medical ward randomly in a 1:1 ratio to either the intervention or usual care…"<br>Comments
**Allocation concealment (selection bias)**	**UNCERTAIN**	Comments the authors gave no information on how the allocation concealment was made, this would potentially bring bias to the study.
**Blinding of participants and personnel (performance bias)**	**HIGH**	Comments According to the nature of the study, it was not possible for the investigators to be blinded to the allocations.
**Blinding of outcome assessment (detection bias)**	**UNCERTAIN**	Comments The authors did not mention whether the assessment was blinded to the results, which would potentially bring bias to the study.
**Incomplete outcome data (attrition bias)**	**LOW**	Comments All the patients' data was reported.
**Selective reporting (reporting bias)**	**LOW**	Comments All the outcomes were reported
**Other bias**	**UNCERTAIN**	Comments the follow-up time was 24-hour, prolonged follow-up time may be required for further evaluation of the outcomes.

Rampon 2022

Bias	Authors' judgement	Support for judgement
**Random sequence generation (selection bias)**	**LOW**	Quote "…Participants were allocated to the prone positioning intervention arm or the usual care arm using response adaptive randomization based on the posterior probability of the intervention being superior to usual care…." Comments the randomization was not likely to bias to the study.
**Allocation concealment (selection bias)**	**HIGH**	Quote "…No concealment or blinding will be used in this study. Participants, study personnel, and clinicians will be aware of treatment assignments…."
**Blinding of participants and personnel (performance bias)**	**HIGH**	Quote "…No concealment or blinding will be used in this study. Participants, study personnel, and clinicians will be aware of treatment assignments…."
**Blinding of outcome assessment (detection bias)**	**UNCERTAIN**	Not mentioned
**Incomplete outcome data (attrition bias)**	**LOW**	Comments All the patients' data was reported.
**Selective reporting (reporting bias)**	**LOW**	Comments All the outcomes were reported
**Other bias**	**HIGH**	Quote "…The study began enrollment on April 25, 2020, and halted enrollment on March 25, 2021, after the study met stopping criteria for low enrollment (one or fewer participants enrolled per week for three consecutive weeks)…" Comments As the study stopped earlier than expected, which was much likely to bring bias to the results.

Rosen 2021

Bias	Authors' judgement	Support for judgement
**Random sequence generation (selection bias)**	**LOW**	Quote "…Randomization allocation was obtained via a centralized web-based system…"<br>Comments centralized web-base system was validated for random sequence generation, and was not likely to bring bias to the study.
**Allocation concealment (selection bias)**	**UNCERTAIN**	Comments The authors gave no information on how the allocation concealment was made, and this would potentially bring bias to the results.
**Blinding of participants and personnel (performance bias)**	**HIGH**	Quote "…Due to the nature of the intervention, the pats, the treating physician, care provider, data collectors and outcome assessors were aware of the allocation…"<br>Comments As stated by the authors, for the design of study, it was not possible to be blinded to the investigators.
**Blinding of outcome assessment (detection bias)**	**HIGH**	As above
**Incomplete outcome data (attrition bias)**	**LOW**	Comments All the patients completed the follow-up with no incomplete outcome data.
**Selective reporting (reporting bias)**	**LOW**	Comments All the outcomes described in the method section was reported in the result section.
**Other bias**	**UNCERTAIN**	Quote "…There are also limitations to this trial. First, the trial was halted early resulting in limited statistical power to detect differences between groups…Forth as all study sites became overwhelmed by severely ill patients with COVID-19, and research staff was relocated for clinical service, we were not able to identify all patients eligible for inclusion…"<br>Comments The performance of the study could potentially bring bias the study.

Tylor 2021

Bias	Authors' judgement	Support for judgement
**Random sequence generation (selection bias)**	**LOW**	***Quote*** "…Five medical admitting teams were randomized using computer-generated random numbers. Teams were randomized in a near 1:1 ratio to deliver UC alone (n = 2 clusters) versus UC plus APPS intervention (n = 3 clusters)…"<br>***Comments*** Random sequence was generated by computer, which was not likely to bring bias to the results.
**Allocation concealment (selection bias)**	**UNCERTAIN**	Comments the authors did not state how the allocation was concealed and this would potentially bring bias to the study.
**Blinding of participants and personnel (performance bias)**	**HIGH**	Quote "…Clinicians were unblinded to treatment allocation, and enrolled patients were considered unblinded…"<br>Comments According to the protocols, the patients were not blinded to the allocations
**Blinding of outcome assessment (detection bias)**	**UNCERTAIN**	Quote "…Clinical and safety outcomes were collected from the electronic health recorded study investigators blinded to treatment assignment…"<br>Comments The randomization was blinded to the investigators. However, whether the analysis process was blinded was not described.
**Incomplete outcome data (attrition bias)**	**LOW**	Comments All the outcomes were reported.
**Selective reporting (reporting bias)**	**LOW**	Comments All the patients' outcomes described in the protocol was reported.
**Other bias**	**HIGH**	Comments As this was a pilot and feasibility study, and patients enrolled in the study was not adhere to the protocols, and this would potentially bring great bias to the results.
