# Supplementary figures and images for: Awake prone position in COVID-19-related acute respiratory failure: a meta-analysis of randomized controlled trials
Source: BMC Pulm Med. 2023 Apr 26;23:145. doi: 10.1186/s12890-023-02442-3 (PMC10131466; doi:10.1186/s12890-023-02442-3)

Additional File 4: Secondary outcomes -- Hospital LOS

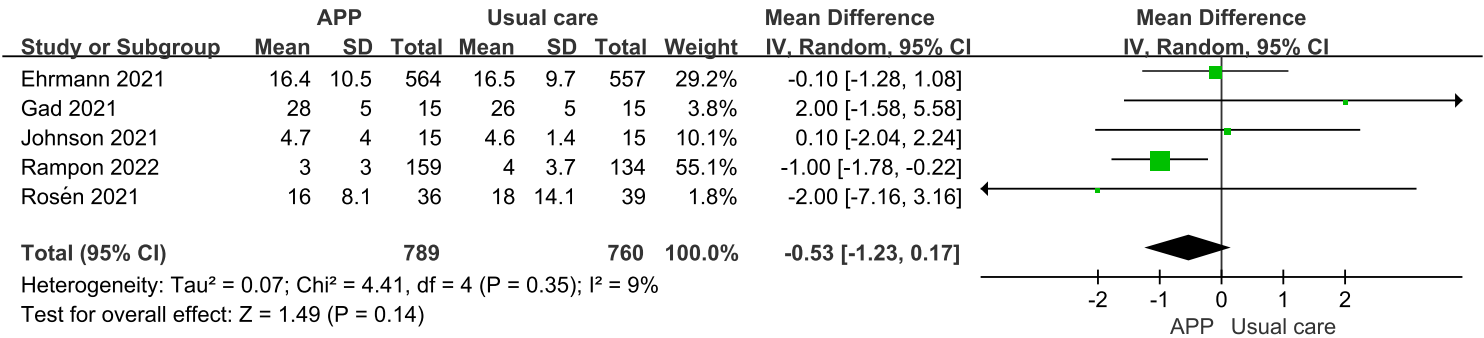

## Additional File 4: Secondary outcomes -- ICU LOS

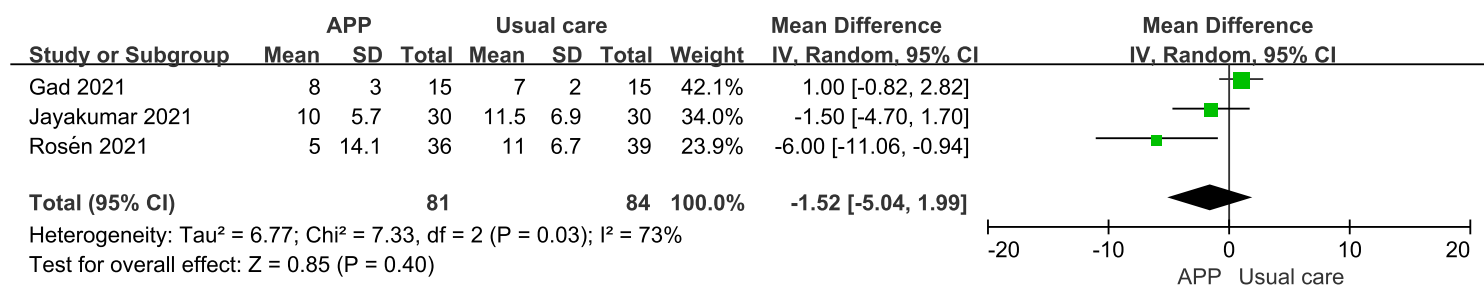

Supplement: Supplementary file 4 — Supplementary Material 4 [file 12890_2023_2442_MOESM4_ESM.pdf]
