# Supplementary material for: Awake prone position in COVID-19-related acute respiratory failure: a meta-analysis of randomized controlled trials
Source: BMC Pulm Med. 2023 Apr 26;23:145. doi: 10.1186/s12890-023-02442-3 (PMC10131466; doi:10.1186/s12890-023-02442-3)
Supplement: Supplementary file 5 — Supplementary Material 5 [file 12890_2023_2442_MOESM5_ESM.pdf]

Additional File 6: Publication bias analyzed by funnel plots

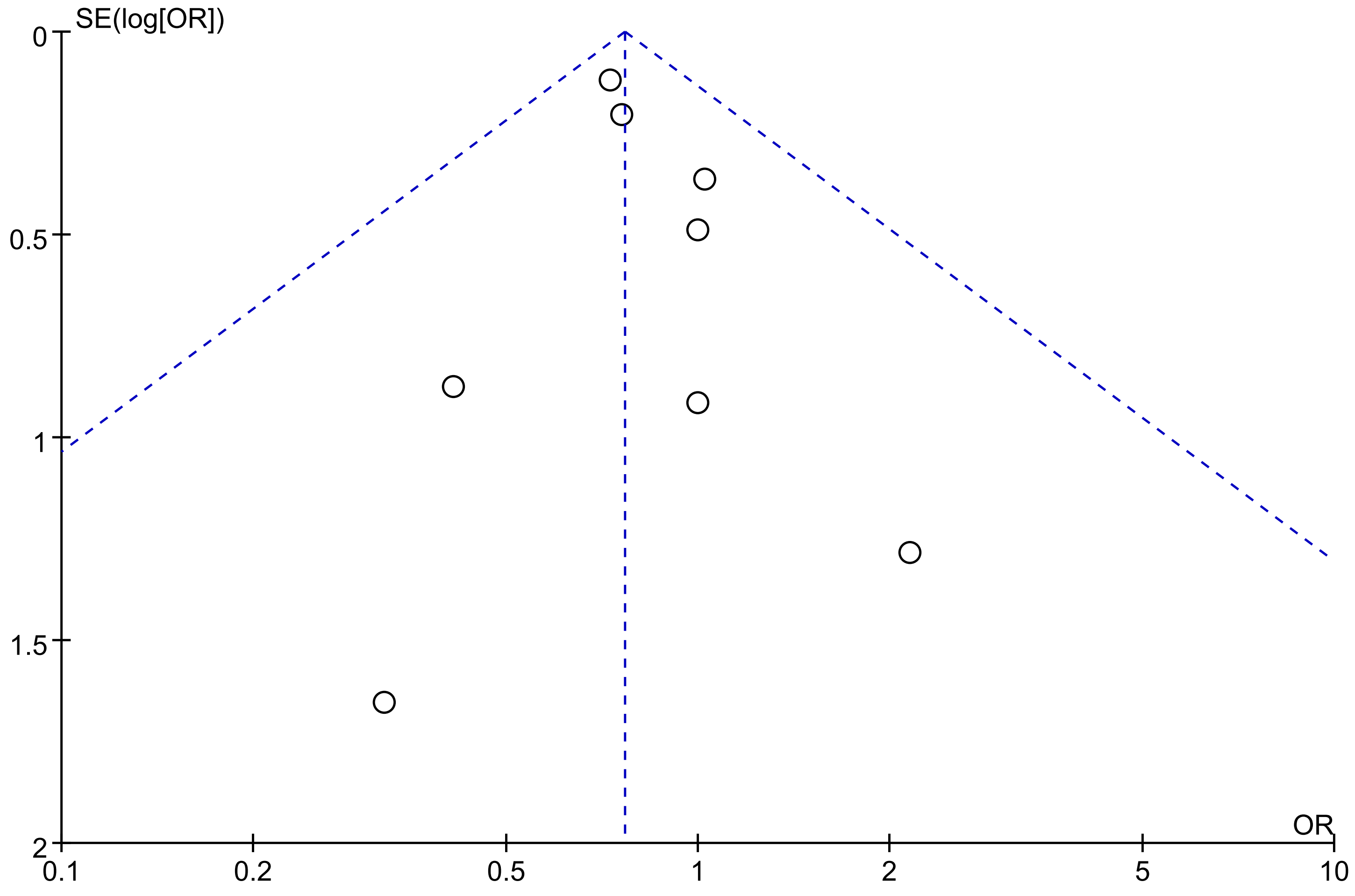

Additional File 6: Publication bias analysis by trim and fill method

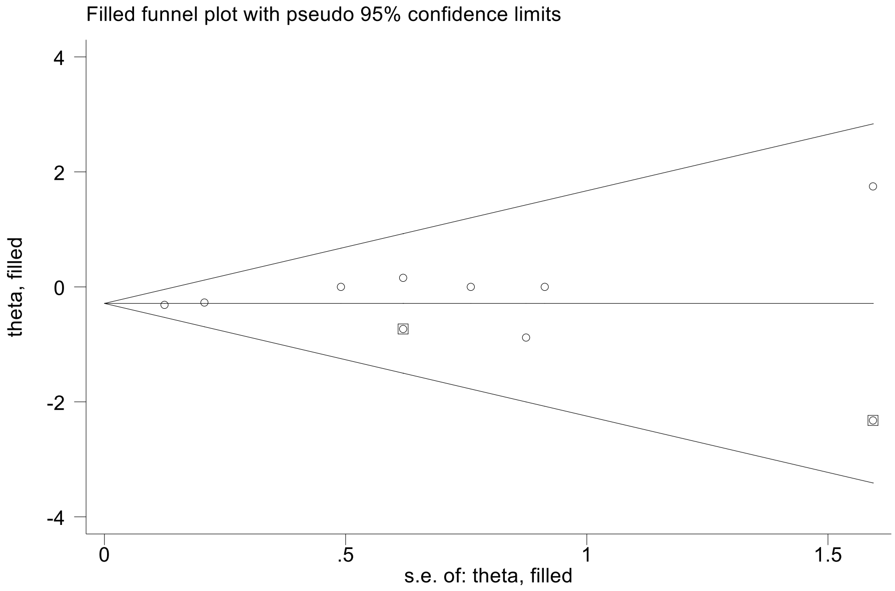

Meta-analysis

	Pooled	95% CI		Asymptotic		No. of
Method	Est	Lower	Upper	z_value	p_value	studies
Fixed	-0.268	-0.465	-0.071	-2.670	0.008	8
Random	-0.268	-0.465	-0.071	-2.670	0.008	

Test for heterogeneity: Q= 3.206 on 7 degrees of freedom (p= 0.865)

Moment-based estimate of between studies variance = 0.000

Trimming estimator: Linear

Meta-analysis type: Fixed-effects model

iteration	estimate	Tn	# to trim	diff
1	-0.268	26	2	36
2	-0.288	27	2	2
3	-0.288	27	2	0

Filled

Meta-analysis

	Pooled	95% CI		Asymptotic		No. of
Method	Est	Lower	Upper	z_value	p_value	studies

-------+---

Fixed | -0.288 -0.482 -0.094 -2.910 0.004 10
