# Supplementary material for: Awake prone position in COVID-19-related acute respiratory failure: a meta-analysis of randomized controlled trials
Source: BMC Pulm Med. 2023 Apr 26;23:145. doi: 10.1186/s12890-023-02442-3 (PMC10131466; doi:10.1186/s12890-023-02442-3)
Supplement: Supplementary file 6 — Supplementary Material 6 [file 12890_2023_2442_MOESM6_ESM.pdf]

Additional File 5: Sensitivity analysis, by omitting each study with the remaining studies re-analyzed

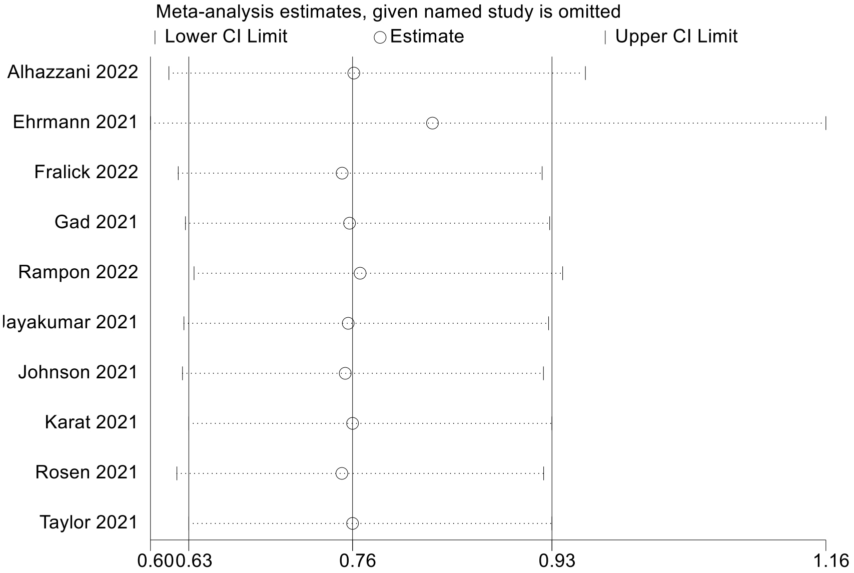

Study omitted	Estimate	[95% Conf.	Interval]
Alhazzani 2022	0.77	0.61	0.96
Ehrmann 2021	0.83	0.60	1.16
Fralick 2022	0.76	0.62	0.92
Gad 2021	0.76	0.63	0.93
Rampon 2022	0.77	0.63	0.94
Jayakumar 2021	0.76	0.62	0.93
Johnson 2021	0.76	0.62	0.92
Karat 2021	0.76	0.63	0.93
Rosen 2021	0.76	0.62	0.92
Taylor 2021	0.76	0.63	0.93
Combined	0.76	0.63	0.93
