# Supplementary material for: Awake prone position in COVID-19-related acute respiratory failure: a meta-analysis of randomized controlled trials
Source: BMC Pulm Med. 2023 Apr 26;23:145. doi: 10.1186/s12890-023-02442-3 (PMC10131466; doi:10.1186/s12890-023-02442-3)
Supplement: Supplementary file 7 — Supplementary Material 7 [file 12890_2023_2442_MOESM7_ESM.pdf]

Additional File 7: Heterogeneity analyzed by Labbe plott

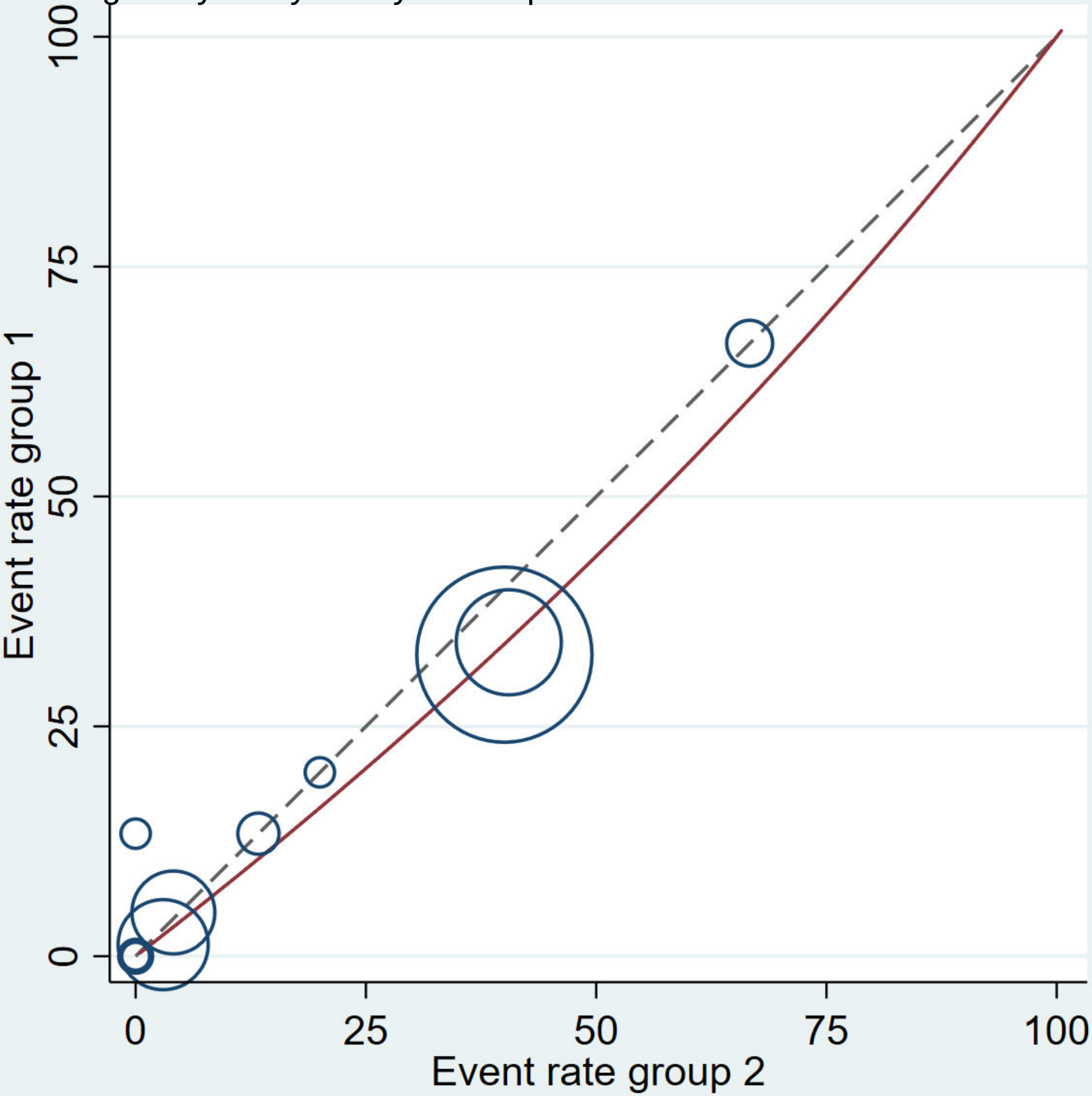

Additional File 7: Heterogeneity analysis by meta-regression

Patient number

Meta-regression Number of obs = 8
REML estimate of between-study variance tau2 = .008292
% residual variation due to heterogeneity I-squared_res = 0.00%
Proportion of between-study variance explained Adj R-squared = 0.0%
With Knapp-Hartung modification

logor	Coef.	Std. Err.	t	P>t	[95% Conf.	Interval]
Pats no	0.00	0.00	-0.79	0.459	-0.0008242	0.0004218
_cons	-0.10	0.24	-0.42	0.687	-0.6765653	0.4773369

Sites

Meta-regression Number of obs = 8
REML estimate of between-study variance tau2 = 0
% residual variation due to heterogeneity I-squared_res = 0.00%
Proportion of between-study variance explained Adj R-squared = 0.0%
With Knapp-Hartung modification

logor	Coef.	Std. Err.	t	P>t	[95% Conf.	Interval]
sites	-0.01	0.01	-0.86	0.421	-0.026138	0.0125074
_cons	-0.05	0.27	-0.18	0.866	-0.7187614	0.6224851

Intubation rate

Meta-regression Number of obs = 8
REML estimate of between-study variance tau2 = 0
% residual variation due to heterogeneity I-squared_res = 0.00%
Proportion of between-study variance explained Adj R-squared = 0.0%
With Knapp-Hartung modification

logor	Coef.	Std. Err.	t	P>t	[95% Conf.	Interval]
Intubation rate	-0.55416	1.09987	-0.5	0.632	-3.24545	2.137122
_cons	-0.11941	0.40815	-0.29	0.78	-1.11811	0.879302

APP time

Meta-regression Number of obs = 6
REML estimate of between-study variance tau2 = 0
% residual variation due to heterogeneity I-squared_res = 0.00%
Proportion of between-study variance explained Adj R-squared = 0.0%
With Knapp-Hartung modification

logor	Coef.	Std. Err.	t	P>t	[95% Conf.	Interval]
APP time	0.0397002	0.12	0.34	0.76	-0.3332885	0.4126889
_cons	-0.4720862	0.61	-0.77	0.50	-2.416794	1.472622

Settings

Meta-regression Number of obs = 8

REML estimate of between-study variance tau2 = 0
% residual variation due to heterogeneity I-squared_res = 0.00%
Proportion of between-study variance explained Adj R-squared = 0.0%
With Knapp-Hartung modification

logor	Coef.	Std. Err.	t	P>t	[95% Conf.	Interval]
Setting ICU	-0.2667216	0.49	-0.54	0.61	-1.47	0.9384186
_cons	-0.0130897	0.48	-0.03	0.98	-1.19	1.165552
